# Resistance Reduction of Conductive Patterns Printed on Textile by Curing Shrinkage of Passivation Layers

**DOI:** 10.3390/mi11060539

**Published:** 2020-05-26

**Authors:** Tomoya Koshi, Ken-ichi Nomura, Manabu Yoshida

**Affiliations:** Sensing System Research Center (SSRC), National Institute of Advanced Industrial Science and Technology (AIST), 1-1-1 Higashi, Tsukuba, Ibaraki 305-8565, Japan; k-nomura@aist.go.jp (K.i.-N.); yoshida-manabu@aist.go.jp (M.Y.)

**Keywords:** e-textiles, conductive pattern, passivation, stretchable conductive ink, screen printing

## Abstract

Directly printing conductive ink on textiles is simple and compatible with the conventional electronics manufacturing process. However, the conductive patterns thus formed often show high initial resistance and significant resistance increase due to tensile deformation. Achieving conductive patterns with low initial resistance and reduced deformation-induced resistance increase is a significant challenge in the field of electronic textiles (e-textiles). In this study, the passivation layers printed on conductive patterns, which are necessary for practical use, were examined as a possible solution. Specifically, the reduction of the initial resistance and deformation-induced resistance increase, caused by the curing shrinkage of passivation layers, were theoretically and experimentally investigated. In the theoretical analysis, to clarify the mechanism of the reduction of deformation-induced resistance increase, crack propagation in conductive patterns was analyzed. In the experiments, conductive patterns with and without shrinking passivation layers (polydimethylsiloxane) cured at temperatures of 20–120 °C were prepared, and the initial resistances and resistance increases due to cyclic tensile and washing in each case were compared. As a result, the initial resistance was reduced further by the formation of shrinking passivation layers cured at higher temperatures, and reduced to 0.45 times when the curing temperature was 120 °C. The cyclic tensile and washing tests confirmed a 0.48 and a 0.011 times reduction of resistance change rate after the 100th elongation cycle (10% in elongation rate) and the 10th washing cycle, respectively, by comparing the samples with and without shrinking passivation layers cured at 120 °C.

## 1. Introduction

Electronic textiles (e-textiles) have received significant attention for the purpose of achieving imperceptible biological information sensing on the human body [1,2,3,4,5,6,7,8,9,10,11]. For e-textiles, the formation of flexible conductive patterns that have low initial resistance and minimal resistance increase due to tensile deformation is a crucial challenge. Prior studies have primarily used two types of formation methods to form conductive patterns on a textile, i.e., weaving/knitting/sewing of conductive yarns, such as silver-coated yarns [12,13,14,15], and printing conductive inks, such as stretchable silver inks [16,17,18]. From the perspective of simplicity of process and compatibility with the conventional electronics manufacturing process, direct printing of conductive ink on a textile has several advantages. However, despite the continued development of new stretchable conductive inks by several research groups [19,20,21], this approach often causes high initial resistance and significant deformation-induced resistance increase. In addition, the conductive fillers contained in the conductive ink, such as silver flakes, are not chemically stable, making it necessary to passivate the conductive patterns. Some previous studies have reported that initial resistance and resistance increase due to deformation of conductive patterns were affected by the formation of passivation layers [22]; however, the passivation itself has not been discussed in depth, and the effects of passivation-layer formation on the resistance changes of patterns remains unclear.

To address this, in this study, the effect of passivation-layer formation on the initial resistance and deformation-induced resistance increase were theoretically and experimentally examined. In particular, the curing shrinkage of passivation layers was explored. When the passivation layers printed on the conductive patterns are cured by heating, curing shrinkage occurs. Then, the conductive patterns are deformed by the shrinking passivation layers, particularly when the respective stiffnesses of the patterns and textile are low (such as the patterns formed with stretchable silver ink on plain weave cotton). In such a case, initial compressive stress occurs in the conductive patterns, and the contact force between the conductive fillers in the patterns must be increased, here, the increased contact force seems to reduce the initial resistance. In addition, the initial compressive stress must prevent crack propagation in the conductive patterns during the deformation (the details are described in Section 2.1). This should also reduce the deformation-induced resistance increase. The resistance reduction of conductive patterns through the curing shrinkage of passivation layers is compatible with other approaches to resistance reduction, such as the development of new conductive inks and printing processes [23].

To investigate the resistance reduction caused by the formation of shrinking passivation layers, in this study, conductive patterns with and without shrinking passivation layers cured at different temperatures were prepared, and the initial resistance and deformation-induced resistance increase were measured and compared. Specifically, to measure the deformation-induced resistance, a cyclic tensile test and cyclic washing test were conducted as simple and complex deformation tests, respectively.

## 2. Materials and Methods

### 2.1. Theoretical Analysis on Reduction of Deformation-Induced Resistance Increase

The reduction of initial resistance caused by the shrinking passivation layers is a simple phenomenon, but the reduction of deformation-induced resistance increase is complex. During tensile deformation, the resistance increase is primarily caused by the following three phenomena: a decrease in contact force between the conductive fillers contained in the conductive ink, a decrease of the electrical path formed with the conductive fillers (a reversible phenomenon without mechanical breaking), and crack propagation in the conductive pattern (an irreversible phenomenon with mechanical breaking). Crack propagation, in particular, has a significant effect on the resistance increase, namely the reduction of crack propagation corresponds to the reduction of deformation-induced resistance increase. Therefore, here, the reduction of crack propagation caused by the shrinking passivation layers was theoretically analyzed based on fracture mechanics.

Stress intensity factor *K* is used in fracture mechanics to predict the crack propagation. When *K* exceeds critical fracture toughness *K*_c_, that is material property, the crack starts to propagate [24,25]. *K* is determined by the sample geometry, size and location of the crack, and applied stress. Critical stress *σ*_c_ is defined as the applied stress when *K* reaches *K*_c_. Therefore, by comparing the values of *σ*_c_ of conductive patterns with and without passivation layers (with identical dimensions of pattern and crack), we can evaluate the reduction of crack propagation. That is, if *σ*_c_ is increased by the formation of passivation layers, then the crack propagation is reduced. In this study, the values of *σ*_c_ were compared for the following four conditions: the conductive pattern without a passivation layer, with a neutral passivation layer, with shrinking passivation layers, and with expanding passivation layers.

**Figure 1:**
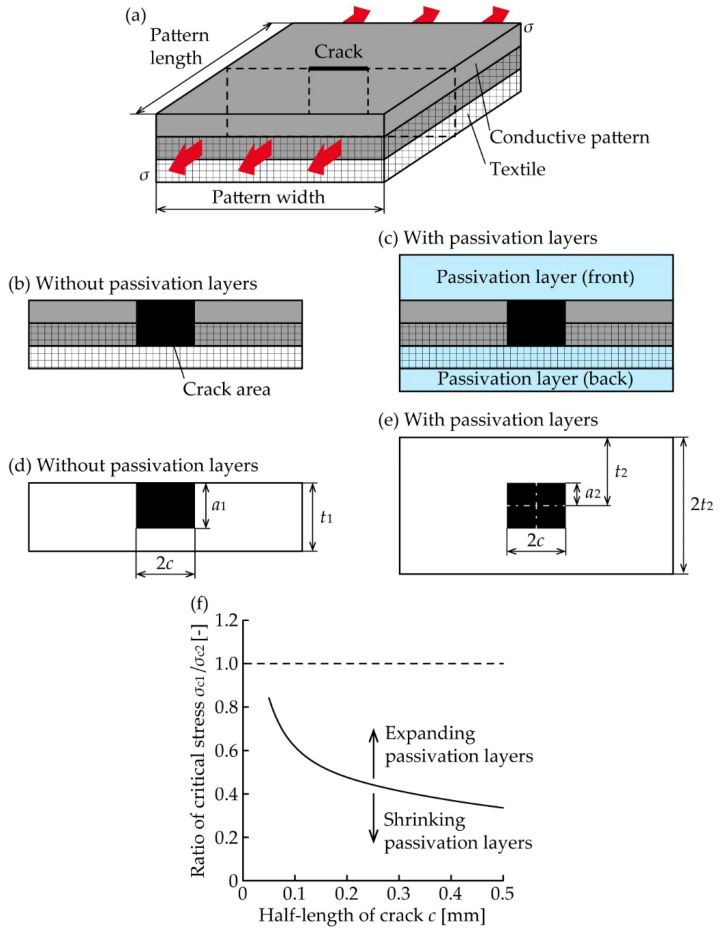
(**a**) Schematic of deformed conductive pattern formed on textile with a small crack. Cross-sectional images of conductive pattern (**b**) without and (**c**) with passivation layers. Parameter definitions of patterns (**d**) without and (**e**) with passivation layers. (**f**) Calculated curve of *σ*_c1_/*σ*_c2_ determined by *c* under identical dimensions of conductive pattern and crack.

Figure 1a illustrates a conductive pattern with formation of a small crack on a textile. The pattern permeates half the thickness of the textile because it is directly formed on the textile through printing. Figure 1b,c, respectively, illustrate the cross-sectional images of the cracked conductive pattern without and with passivation layers shown in Figure 1a. For simplicity, there is no delamination between each layer, and the mechanical properties and thickness of each layer are identical. In this case, we can regard the cracked conductive patterns without and with passivation layers as finite plates with surface and embedded cracks, respectively. Therefore, the stress intensity factors for mode I (crack opening mode) *K*_I_ are given by [26]:

(1) $$\begin{array}{cc} K_{I1}=Y_{1}\sigma_{1}\sqrt{{\pi a}_{1}} & \left(\mathrm{for}\;\mathrm{without}\;\mathrm{passivation}\;\mathrm{layers}\right) \end{array}$$

(2) $$\begin{array}{cc} K_{I2}=Y_{2}\sigma_{2}\sqrt{{\pi a}_{2}} & \left(\mathrm{for}\;\mathrm{without}\;\mathrm{passivation}\;\mathrm{layers}\right) \end{array}$$

where *Y*, *σ*, and *a* are dimensionless factor depending on the sample geometry, remote uniform tensile stress, and crack depth, respectively (Figure 1d,e). Subscripts 1 and 2 correspond to patterns without and with passivation layers. When we use the equations for elliptical crack [26], *Y*_1_ and *Y*_2_ are given by

(3) $$Y_{1}=\left[M_{11}+M_{12}{\left(\frac{a_{1}}{t_{1}}\right)}^{2}+M_{13}{\left(\frac{a_{1}}{t_{1}}\right)}^{4}\right]/\sqrt{Q_{1}}$$

(4) $$Y_{2}=\left[M_{21}+M_{22}{\left(\frac{a_{2}}{t_{2}}\right)}^{2}+M_{23}{\left(\frac{a_{2}}{t_{2}}\right)}^{4}\right]/\sqrt{Q_{2}}$$

respectively, where

(5) $$M_{11}=1$$

(6) $$M_{12}=\frac{0.05}{0.11+{\left(\frac{a_{1}}{c}\right)}^{3/2}}$$

(7) $$M_{13}=\frac{0.29}{0.23+{\left(\frac{a_{1}}{c}\right)}^{3/2}}$$

(8) $$Q_{1}=1+1.464{\left(\frac{a_{1}}{c}\right)}^{1.65}$$

(9) $$M_{21}=1.13-0.09\left(\frac{a_{2}}{c}\right)$$

(10) $$M_{22}=-0.54+\frac{0.89}{0.2+\frac{a_{2}}{c}}$$

(11) $$M_{23}=0.5-\frac{1}{0.65+\frac{a_{2}}{c}}+14{\left(1-\frac{a_{2}}{c}\right)}^{24}$$

(12) $$Q_{2}=1+1.464{\left(\frac{a_{2}}{c}\right)}^{1.65}$$

Furthermore, *t* and *c* are the thickness or half-thickness of the plate and half-length of the crack, respectively. Note that Equations (1)–(12) satisfy *a*_1_/*c* < 1 and *a*_2_/*c* < 1, and *c* is much smaller than the pattern width. The ratio of critical stresses *σ*_c1_ and *σ*_c2_, that satisfy *K*_I1_ = *K*_I2_ = *K*_c_, respectively, is given by

(13) $$\frac{\sigma_{c1}}{\sigma_{c2}}=\frac{Y_{2}\sqrt{a_{2}}}{Y_{1}\sqrt{a_{1}}}$$

Figure 1f shows the calculated curve of *σ*_c1_/*σ*_c2_ determined by *c* under identical dimensions of the conductive pattern and crack. In the calculation, the thickness of the conductive pattern was 50 μm; total thicknesses of patterns without and with passivation layers were 75 and 150 μm (*t*_1_ = 75 µm and *t*_2_ = 75 µm (2*t*_2_ = 150 μm)), respectively; crack depth was 50 μm, (*a*_1_ = 50 µm and *a*_2_ = 25 µm (2*a*_2_ = 50 μm). Figure 1f confirms that *σ*_c1_/*σ*_c2_ decreased as *c* increased and was always lower than 1.0. That is, *σ*_c2_ is always greater than *σ*_c1_ even when the crack dimension shows a high or low aspect (width/depth = 2:1 or 20:1, respectively). This indicates that the crack propagation is reduced by the formation of neutral passivation layers. For the shrinking passivation layers, the initial compressive stress is caused in the conductive pattern, and therefore the stress at the identical elongation of conductive pattern decreases. As a result, *σ*_c2_ relatively increases and the curve of *σ*_c1_/*σ*_c2_ shifts to lower values. This indicates that the crack propagation is reduced further by the formation of shrinking passivation layers than by the neutral layers. For the expanding passivation layers, the curve of *σ*_c1_/*σ*_c2_ shifts to greater values because of the occurrence of the initial tensile stress. In this case, only when *σ*_c1_/*σ*_c2_ remains lower than 1.0, the formation of expanding passivation layers also reduces the crack propagation.

### 2.2. Sample Preparation

A stretchable silver ink, polydimethylsiloxane (PDMS), and plain weave cotton were used for the conductive patterns, passivation layers, and textiles, respectively. Because stretchable silver ink has high flexibility and a low Young’s modulus, it does not degrade the flexibility of the textile [27]. PDMS is a material that is commonly used across research fields; it has high flexibility, insulation property, chemical stability, and biocompatibility. Plain weave cotton is one of the most popular textiles in the industry and is easily available.

**Figure 2:**
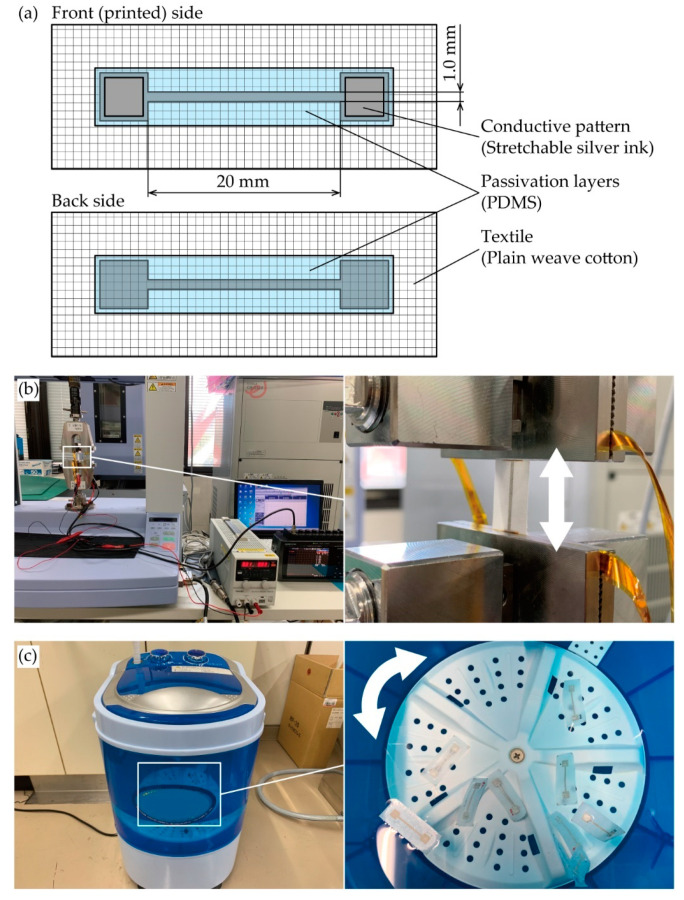
(**a**) Schematic of prepared sample. Optical images of experimental setups of (**b**) cyclic tensile test and (**c**) cyclic washing test.

The viscosity of the stretchable silver ink (SSP2801, Toyobo Co., LTD., Osaka, Japan) was 20.5 Pa·s at a shear rate of 100 s^−^^1^. The diameter and pitch of the commercially available plain weave cotton yarns were 0.2 mm and 0.3 mm, respectively, and the plain weave thickness was 0.22 mm. Figure 2a shows the schematic of the conductive pattern. The pattern had a straight shape with a pair of contact pads, and the designed width and length were 1.0 and 20 mm, respectively. The pattern was designed based on the samples used for resistance measurements under tensile deformation in previous studies [19,28,29].

The samples with and without passivation layers were prepared as follows. First, the ink was screen-printed on a piece of plain weave cotton. Then, the samples were heated at 120 °C for 30 min using a hot plate to cure the conductive pattern. Next, for the samples with passivation layers, a liquid-state PDMS (Sylgard 184 silicone elastomer kit, Dow Corning, Midland, MI, USA), consisting of a base and curing agent mixed in weight ratios of 10:1 (base/curing agent), was screen-printed on the back of the conductive pattern. Then, the PDMS layer was cured at 20 (room temperature (RT)), 40, 60, 80, 100, and 120 °C for 48 h, 16 h, 4 h, 2 h, 80 min, and 40 min, respectively. In this case, the PDMS demonstrated both curing expansion and shrinkage [30]. Following this, liquid-state PDMS was also screen-printed on the front (printed side) of the conductive pattern, and it was cured under the same conditions as for the back of the PDMS layer. As each PDMS layer was cured, samples without the passivation layers were simultaneously heated, because the initial resistance of conductive patterns is also affected by the heating temperature and its total time of application to the pattern. Here, the heating conditions of conductive patterns with and without passivation layers were made identical in order to exclusively examine the effect of passivation-layer formation on the resistance change. The relationship between the curing temperature *T*_c_ and the heating temperature and its total time of application to each layer is shown in Table 1.

### 2.3. Experimental Setup

For the measurement of initial resistance, the resistance of the conductive patterns with and without passivation layers were measured with a source meter (2400, Keithley Co. Ltd., Cleveland, OH, USA) using a four-terminal method. Figure 2b,c shows the experimental setups of the cyclic tensile and washing tests. For the cyclic tensile test, a prepared sample was mounted on a tensile testing machine (AGS-X, Shimadzu Co., Kyoto, Japan), and cyclic tensile deformations were applied to it. The elongation rate, number of cycles, and elongation speed rate were 10%, 100 cycles, and 17 mm/min, respectively. During the test, the constant current of 10 mA was applied to the conductive pattern with a constant current power supply (PA36-2B, Texio Technology Co., Kanagawa, Japan), and the voltage between the contact pads of pattern was measured by a data logger (GL900-APS, Graphtec Co., Kanagawa, Japan) using a four-terminal method. The resistance of the conductive pattern was calculated by dividing the measured voltage by the current. For the cyclic washing test, the prepared samples were put into a washing machine (MWM1000, Mitsukin Shoji Co. Ltd., Oita, Japan) and washed with tap water for 9 min. During the washing, the direction of water rotation was regularly changed between right and left. Following the washing, the samples were naturally dried at RT. After drying, the resistance of the conductive pattern was measured with a source meter (2400, Keithley Co. Ltd., Cleveland, OH, USA) using a four-terminal method.

## 3. Results and Discussion

### 3.1. Reduction of Initial Resistance by Formation of Passivation Layers

**Figure 3:**
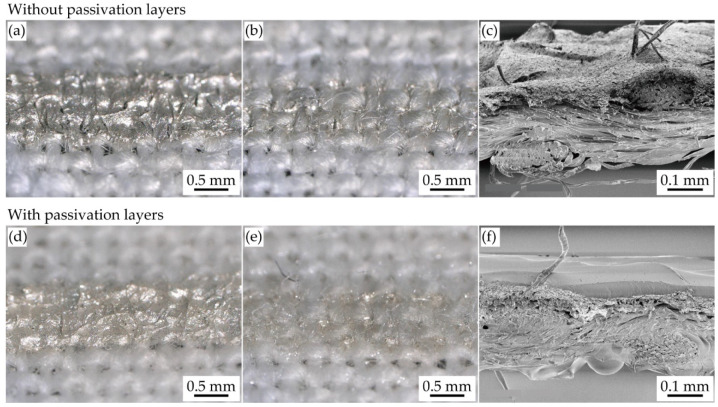
Optical images of (**a**) front (printed) and (**b**) back side, and (**c**) cross-sectional SEM image of conductive pattern without passivation. Optical images of (**d**) printed and (**e**) back side, and (**f**) cross-sectional SEM image of conductive pattern with passivation (*T*_c_ = 120 °C).

Before measuring the reduction of initial resistance due to the formation of shrinking passivation layers cured at different temperatures, the prepared samples were observed with an optical microscope and scanning electron microscope (SEM) (VHX-D510, Keyence Co., Osaka, Japan), as shown in Figure 3a–f. Figure 3a–f shows the optical and SEM images of a conductive pattern without and with the shrinking passivation layers cured at 120 °C, respectively. Figure 3b,e confirms that the printed stretchable silver ink permeates from the front to the back of the textile in the cases both with and without passivation. The measured conductive pattern width was 1.2 mm, although the designed value was 1.0 mm. This is because of the deep permeation of ink into the textile. The cross-sectional images of the conductive pattern without and with the passivation are shown in Figure 3c,f, respectively. Figure 3f confirms that the conductive pattern is completely covered with the passivation layers, and the effective thicknesses of the conductive pattern, front passivation layer, and back passivation layer are approximately 50, 40, and 50 μm, respectively.

**Figure 4:**
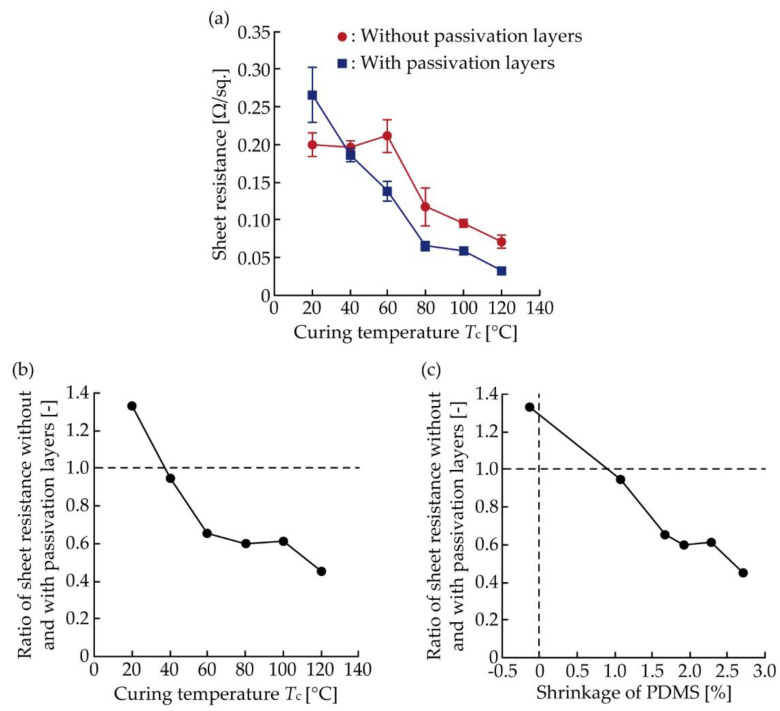
(**a**) Average sheet resistance of conductive pattern without and with passivation determined by *T*_c_; (**b**) Ratio of average sheet resistance without and with passivation, determined by *T*_c_; (**c**) Ratio of average sheet resistance without and with passivation, determined by PDMS shrinkage.

Figure 4a shows the average initial sheet resistance of conductive patterns with and without passivation layers cured at different temperatures. In Figure 4a, the initial sheet resistance was calculated by dividing the measured resistance by the pattern length (20 mm) and producing the pattern width (1.2 mm). The number of samples used for each plot was nine. For the conductive pattern without passivation layers, the sheet resistance had an almost consistent value (approximately 0.2 Ω/sq.) when *T*_c_ ranged between 20–60 °C, and then decreased as *T*_c_ increased from 60 to 120 °C. This was because the total heat applied to the conductive layer increased, as shown in Table 1, and the contact force between the conductive fillers in the conductive ink is expected to have increased based on the shrinkage of organic binder contained in the ink. For the conductive patterns with passivation layers, the sheet resistance gradually decreased from 0.27 to 0.032 Ω/sq. as *T*_c_ increased from 20 to 120 °C. Figure 4b shows the ratio of sheet resistance without and with passivation. The ratio was calculated by dividing the average sheet resistance without passivation by that with passivation from Figure 4a. The ratio decreased from 1.33 to 0.45 as *T*_c_ increased from 20 to 120 °C. Figure 4c shows the ratio of sheet resistance with and without passivation, determined by the shrinkage of PDMS. The shrinkage was converted from *T*_c_ based on a previous study [30]. In Figure 4c, the positive and negative values of shrinkage indicate the shrinking and expansion, respectively. The ratio was always lower than 1.0, except for when *T*_c_ was 20 °C (expansion). Therefore, Figure 4b,c confirms that the initial resistance was reduced by the formation of shrinking passivation layers. In addition, Figure 4c confirms that the ratio of sheet resistance decreased almost linearly as the shrinkage increased. This indicates that the initial resistance can be further reduced by the formation of more shrinking passivation layers.

### 3.2. Reduction of Resistance Increase by Formation of Passivation Layers During Cyclic Tensile Test

**Figure 5:**
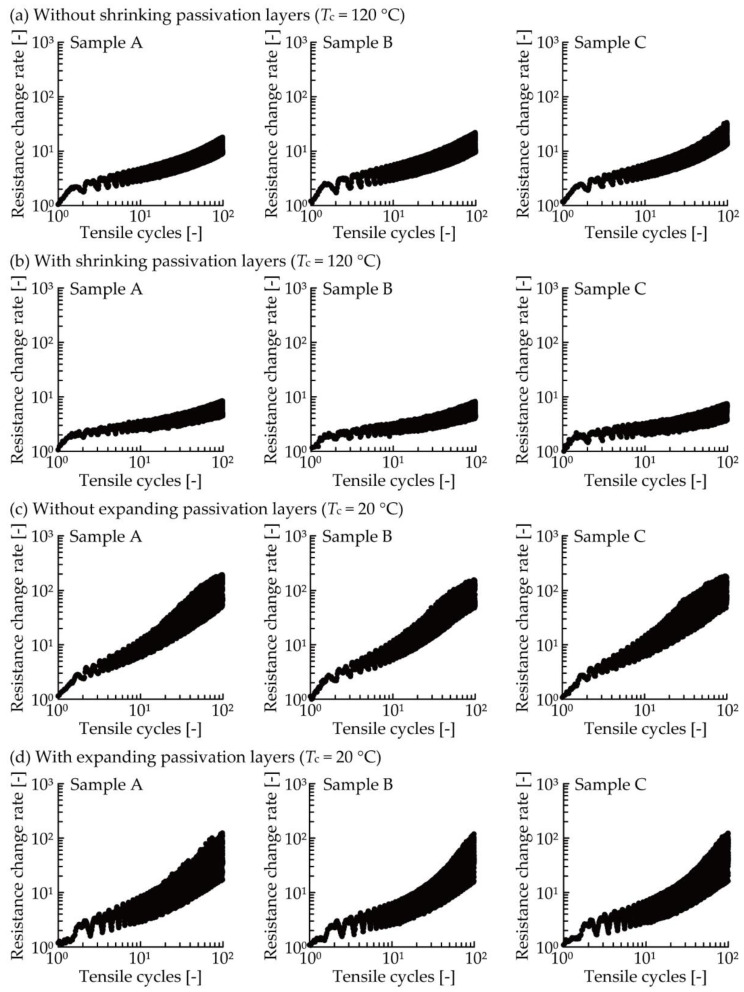
Resistance increase of conductive patterns (**a**) without and (**b**) with shrinking passivation layers (*T*_c_ = 120 °C) during the cyclic tensile test. Resistance increase of conductive pattern (**c**) without and (**d**) with expanding passivation layers (*T*_c_ = 20 °C) during the cyclic tensile test.

**Figure 6:**
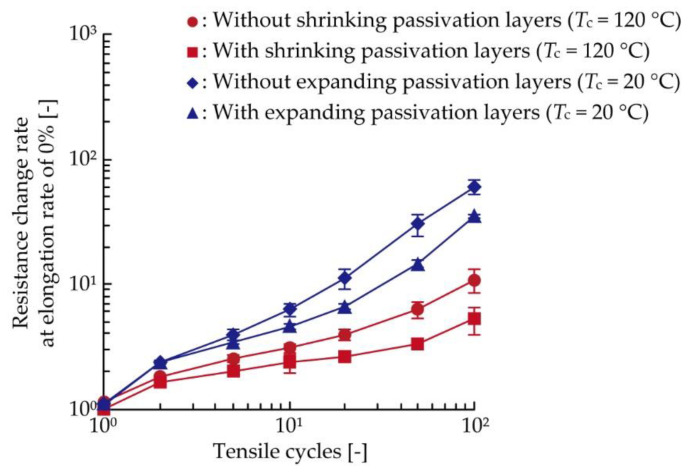
Average value of the resistance change rate at a 0% elongation rate during the 1st, 2nd, 5th, 10th, 20th, 50th, and 100th cycles, shown in Figure 5.

Figure 5a,b shows the resistance increase due to cyclic tensile deformation in the cases of the conductive patterns without and with shrinking passivation layers cured at 120 °C, respectively. There were three samples (samples A, B, and C) under each condition. The resistance change rate was calculated by dividing the resistance by the initial resistance of the conductive pattern. In Figure 5a,b, the resistance repeatedly increased and decreased during each cycle, and the resistance change rate gradually shifted to the higher values as the tensile cycle increased. Comparing Figure 5a,b, the plots in Figure 5b show lower values than those in Figure 5a. For an improved understanding of the reduction of resistance increase illustrated in Figure 5a,b, the resistance increases were numerically compared, as shown in Figure 6. The figure shows the average values of the resistance change rate at an elongation rate of 0% during the 1st, 2nd, 5th, 10th, 20th, 50th, and 100th cycle, respectively. Comparing the plots of conductive patterns without and with the shrinking passivation layers cured at 120 °C (red circle and square plots), the values at 0% during the 100th cycle were reduced from 10.8 to 5.2, a 0.48 times reduction. This confirms that the resistance increase due to cyclic tensile deformation was reduced by the formation of shrinking passivation layers.

In this test, the change of resistance increase due to deformation caused by the expanding passivation layers was also examined. Figure 5c,d shows the resistance increase due to the cyclic tensile deformation in the case of the conductive pattern without and with the expanding passivation layers cured at 20 °C, respectively. In Figure 5c,d, the resistance change rate also increased as the tensile cycle increased, and the resistance of conductive patterns with the expanding passivation layers shows lower values than those without the passivation. In the numerical comparison, shown in Figure 6, it can be seen that the resistance change rate at 0% during the 100th cycle reduced from 60.3 to 35.2, a 0.58 times reduction, due to the formation of expanding passivation layers. This confirms that the resistance increase due to cyclic tensile deformation was also reduced by the formation of expanding passivation layers under the conditions used in this study.

**Figure 7:**
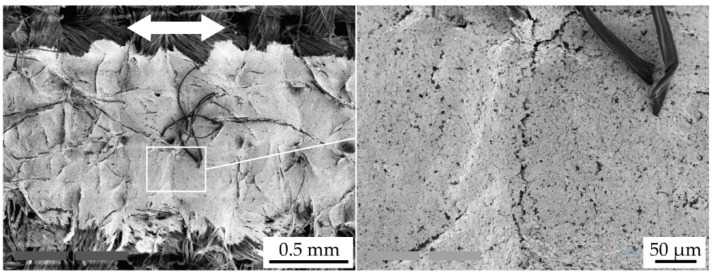
SEM images of crack propagation in the conductive pattern without passivation layers (*T*_c_ = 120 °C) following the cyclic tensile test.

Figure 7 shows the SEM images of the conductive pattern without the shrinking passivation layers, cured at 120 °C, following the cyclic tensile test. Figure 7 confirms that the crack propagation in the conductive pattern was caused by the cyclic tensile deformation. The crack gap was approximately several micrometers, and cracks propagated almost perpendicular to the tensile deformation. This confirms that the resistance increase, shown in Figure 5a,b, was caused by the crack propagation. The initial compressive stress in the conductive pattern, caused by the shrinking passivation layer, must prevent the crack propagation, as described in Section 2.1. Therefore, the deformation-induced resistance increase was reduced by the formation of shrinking passivation layers.

### 3.3. Reduction of Resistance Increase by Formation of Passivation Layers During Cyclic Washing Test

**Figure 8:**
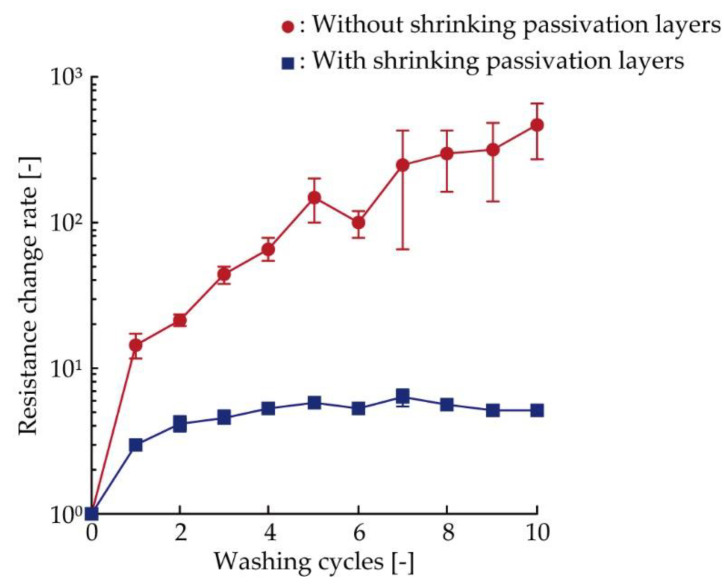
Resistance increase of conductive patterns with and without shrinking passivation layers (*T*_c_ = 120 °C) during the cyclic washing test.

Figure 8 shows the resistance increase due to the washing in the cases of the conductive pattern without and with the shrinking passivation layers cured at 120 °C, respectively. There were three samples under each condition. The resistance change rate was calculated by dividing the resistance after each washing cycle by the initial resistance before the washing test. On the one hand, for the conductive patterns without the shrinking passivation layers, the resistance change rate increased as the washing cycle increased and reached 464 after the 10th washing cycle. On the other hand, for the conductive patterns with shrinking passivation layers, the resistance change rate increased from 1 to 5.8 up to the fifth cycle, but it retained an almost consistent value after five cycles. Comparing the conductive patterns with and without the shrinking passivation layers, the resistance change rate after ten cycles was reduced to 0.011 times. This confirms that the resistance increases due to washing, which is more complex than cyclic tensile deformation, was also reduced by the formation of shrinking passivation layers.

**Figure 9:**
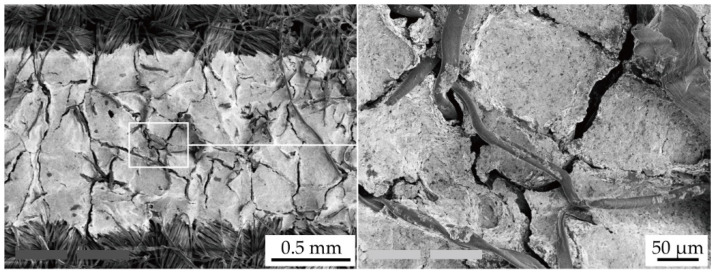
SEM images of crack propagation in the conductive pattern without passivation layers (*T*_c_ = 120 °C) following the cyclic washing test.

Figure 9 shows SEM images of the conductive pattern without the shrinking passivation layers cured at 120 °C following the cyclic washing test. Figure 9 confirms that the deformation during the cyclic washing test caused the crack propagation in the conductive pattern. The direction of crack propagation was not uniform, and the crack gap was several tens of micrometers; therefore, the resulting cracks were larger than those from the cyclic tensile test, as shown in Figure 7. This indicates that the resistance increase in Figure 8 was caused by the crack propagation, and the deformations applied to the conductive patterns during the cyclic washing test were larger and more complex than those in the cyclic tensile test.

## 4. Conclusions

This study focused on the resistance reduction of conductive patterns directly printed on textiles through curing shrinkage of printed passivation layers. The reduction of initial resistance and increase in deformation-induced resistance were theoretically and experimentally investigated. The theoretical analysis explained the mechanism of the reduction of deformation-induced resistance increase by analyzing crack propagation in conductive patterns. In the experiments, conductive patterns with and without shrinking passivation layers, cured at temperatures of 20–120 °C, were prepared, and the initial resistance and increase in resistance due to cyclic tensile and washing deformations were compared. The results showed that the initial resistance was further reduced by the formation of shrinking passivation layers cured at higher temperatures, and the initial resistance was reduced to 0.45 times when *T*_c_ was 120 °C. By comparing the samples with and without shrinking passivation layers cured at 120 °C in the cyclic tensile and washing tests, it could be confirmed that the resistance change rate reduced to 0.48 and 0.011 times after the 100th elongation cycle (10% in elongation rate) and 10th washing cycle, respectively. Therefore, this study demonstrates the usefulness of the formation of shrinking passivation layers in reducing initial resistance and deformation-induced resistance increase in e-textiles.

## Figures and Tables

**Table 1:** Heating temperature and its total time of application to each layer of prepared samples.

Curing Temperature *T*_c_	Heating Temperature and Its Total Time of Application to Each Layer		
	Conductive Layer	Passivation Layer (Front Side)	Passivation Layer (Back Side)
20 °C (RT) (expanding)	120 °C 30 min + 20 °C 48 h + 20 °C 48 h	20 °C 48 h	20 °C 48 h + 20 °C 48 h
40 °C (shrinking)	120 °C 30 min + 40 °C 16 h + 40 °C 16 h	40 °C 16 h	40 °C 16 h + 40 °C 16 h
60 °C (shrinking)	120 °C 30 min + 60 °C 4 h + 60 °C 4 h	60 °C 4 h	60 °C 4 h + 60 °C 4 h
80 °C (shrinking)	120 °C 30 min + 80 °C 2 h + 80 °C 2 h	80 °C 2 h	80 °C 2 h + 80 °C 2 h
100 °C (shrinking)	120 °C 30 min + 100 °C 80 min + 100 °C 80 min	100 °C 80 min	100 °C 80 min + 100 °C 80 min
120 °C (shrinking)	120 °C 30 min + 120 °C 40 min + 120 °C 40 min	120 °C 40 min	120 °C 40 min + 120 °C 40 min
